# Community-Associated MRSA Infection in Remote Amazon Basin Area, Peru

**DOI:** 10.3201/eid2205.151881

**Published:** 2016-05

**Authors:** Coralith García, Lizeth Astocondor, Jinnethe Reyes, Lina P. Carvajal, Cesar A. Arias, Carlos Seas

**Affiliations:** Universidad Peruana Cayetano Heredia, Lima, Peru (C. García, L. Astocondor, C. Seas);; Hospital Nacional Cayetano Heredia, Lima (C. García, C. Seas);; Universidad El Bosque, Bogotá, Colombia (J. Reyes, L.P. Carvajal, C.A. Arias);; University of Texas Medical School at Houston, Houston, Texas, USA (C.A. Arias)

**Keywords:** Community-associated methicillin-resistant Staphylococcus aureus, MRSA, SCC*mec* V, skin and soft tissue infection, bacteria, Amazon Basin, Peru, antimicrobial resistance

**To the Editor:** Two predominant community-associated methicillin-resistant *Staphylococcus aureus* (CA-MRSA) clones have been reported in South America: 1) sequence type 30 staphylococcal cassette chromosome *mec* IV (ST30-SCC*mec *IV) (USA 1100), first found in Uruguay (2002) and later in Brazil and Argentina (2005); and 2) ST8-SCC*mec* IVc/E (USA300–Latin American variant), found predominantly in Ecuador and Colombia (2006–2008) (*1*). In hospitals in Colombia, USA300–Latin American variant has replaced the most common hospital-associated lineage, known as the Cordobes/Chilean clone (MRSA ST5-SCC*mec* I) (*2*). In Peru, a limited number of imported cases of CA-MRSA have been reported (*3*). We describe a case of CA-MRSA infection in a patient living in a remote area of the Amazon Basin of Peru. 

The patient was a 58-year-old woman who was hospitalized in June 2014 for a skin ulcer. She had been well until 10 days before admission, when she noticed a papule on her right arm, followed the next day by localized swelling and redness. Three days later, spontaneous secretion of a purulent material was noted. At the time of admission, the patient had no fever or constitutional symptoms; the ulcer was deep with irregular borders (≈10 × 4 cm) and active purulent secretion (Figure, panel A). Other physical examination findings were unremarkable. 

**Figure:**
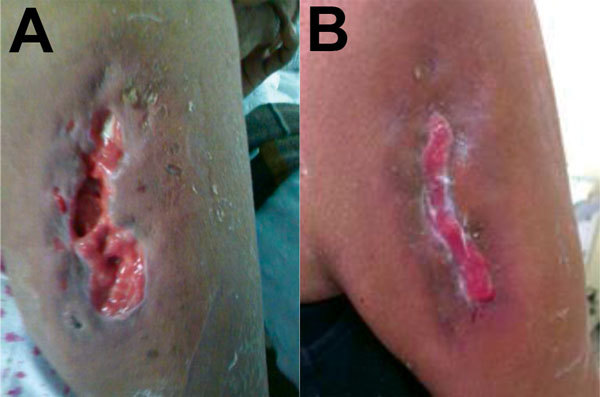
A) Untreated community-associated methicillin-resistant *Staphylococcus aureus* ulcer on the right arm of a 58-year old woman from a rural area of the Amazon Basin, Peru. B) The same ulcer after 19 days of treatment with vancomycin and trimethoprim/sulfamethoxazole.

For the past 2 years, the patient had lived in a remote, rural, jungle village in Peru. She was a housewife but also farmed in nearby areas. There were ducks, chickens, and guinea pigs on the farm where she lived. Her village had neither running water nor roads and almost no access to healthcare (reaching the nearest healthcare center required a 36-hour boat trip). She previously experienced several episodes of malaria (most recently in February 2014), for which she received antimalarial medication provided by a Brazilian military post at the border of Peru. She had never taken antimicrobial drugs and had not traveled in the past 2 years. She first noticed the skin lesion on the first day of an 8-day boat trip from her home village to Iquitos, the largest city in the Peruvian Amazon Basin. 

Cultures from wound exudate and skin biopsy samples yielded *S. aureus* resistant to oxacillin, tetracycline, and erythromycin and susceptible to ciprofloxacin, gentamicin, rifampin, and trimethoprim/sulfamethoxazole (susceptibility testing performed by an agar dilution method). D test showed inducible resistance to clindamycin. The presence of *mecA* and the genes (*lukS-PV*, *lukF-PV*) encoding Panton-Valentine leucocidin (PVL) were confirmed by PCR.

The isolate was characterized as MRSA ST6-t701-SCC*mec*V. Whole-genome sequence analyses identified a predicted protein with 100% aa identity (98% coverage) to the truncated β-hemolysin of the reference genome of *S. aureus* USA300_FPR3757 (GenBank accession no. gb|ABD20946.1|) and the prophage groups 1, 2, and 3. Pulsed-field gel electrophoresis (PFGE) exhibited a pulsotype different from other typical CA-MRSA PFGE patterns found in MRSA from Latin America, labeled as CA-MRSA 120 (Technical Appendix). Intravenous clindamycin (600 mg every 8 hours for 5 days) was empirically prescribed, after which treatment was switched to vancomycin (1 g every 12 hours for 1 week). Subsequently, the patient received trimethoprim/sulfamethoxazole (160/800 mg every 12 hours for 1 week). The clinical evolution was satisfactory, and the infection resolved (Figure, panel B).

This case of a skin and soft tissue infection caused by a CA-MRSA ST6-t701-SCC*mec* V PVL-producing organism is notable for several reasons. First, infection occurred in a remote rural jungle area of Peru at the border with Brazil and Colombia and resembles the first cases of CA-MRSA described in the early 1990s as occurring in indigenous people living in remote areas of Western Australia (*4*). Second, considering that the most predominant CA-MRSA clones in Latin America carry SCC*mec* IV (*1*,*5*), finding SCC*mec* V in this isolate was not expected. MRSA carrying SCC*mec* V have been well characterized as colonizers and agents of infection in animals and in humans in close contact with animals (mainly in Europe but also in other parts of the world) (*6*). These livestock-associated MRSA clones predominantly belong to ST97 (which are usually not PVL producers) and ST398. In addition, ST398 SCC*mec *V MRSA isolates from pigs in Peru have been described (*7*). Of note, methicillin-susceptible *S. aureus* t701 and MRSA t701 carrying SCC*mec* II have recently been found in China, isolated from patients during food poisoning outbreaks and from colonized pork butchers, respectively (*8*,*9*). In South America, isolation of non–PVL-producing MRSA t701 (carrying SCC*mec* IVc) and methicillin-sensitive S*. aureus* t701 from colonized inpatients has been well described (*10*). Although speculation that animal carriage might have played a role in this infection is tempting, further studies are needed to recognize the origin of this MRSA ST6-SCC*mec* V PVL producer in this area of the Amazon Basin. 

**Technical Appendix.** Pulsed-field gel electrophoresis of known hospital-associated and community-associated methicillin-resistant *Staphylococcus aureus* isolates from Latin America.

## References

[1] Reyes J, Rincón S, Díaz L, Panesso D, Contreras GA, Zurita J, Dissemination of methicillin-resistant *Staphylococcus aureus* USA300 sequence type 8 lineage in Latin America. Clin Infect Dis. 2009;49:1861–7. 10.1086/648426 19911971 PMC2787674

[2] Reyes J, Arias C, Carvajal L, Rojas N, Ibarra G, Garcia C, MRSA USA300 variant has replaced the Chilean clone in Latin American hospitals. 2012. Abstract presented at: the Interscience Conference on Antimicrobial Agents and Chemotherapy; 2012 Sep 9–12; San Francisco, CA, USA.

[3] García C, Deplano A, Denis O, León M, Siu H, Chincha O, Spread of community-associated methicillin-resistant *Staphylococcus aureus* to Peru. J Infect. 2011;63:482–3. 10.1016/j.jinf.2011.09.001 21920381

[4] Udo EE, Pearman J, Grub W. Genetic analysis of community isolates of methicillin-resistant *Staphylococcus aureus* in Western Australia. J Hosp Infect. 1993;25:97–108. 10.1016/0195-6701(93)90100-E 7903093

[5] Ma XX, Galiana A, Pedreira W, Mowszowicz M, Christophersen I, Machiavello S, Community- acquired methicillin-resistant *Staphylococcus aureus*, Uruguay. Emerg Infect Dis. 2005;11:973–6. 15963301 10.3201/eid1106.041059 PMC3367603

[6] Spoor LE, Mcadam PR, Weinert LA, Rambaut A, Hasman H, Aarestrup FM, Livestock origin for a human pandemic clone of community-associated methicillin-resistant *Staphylococcus aureus.* MBio. 2013;4:1–6 . 10.1128/mBio.00356-13 23943757 PMC3747577

[7] Arriola CS, Guere M, Larsen J, Skov RL, Gilman RH, Armando E, Presence of methicillin-resistant *Staphylococcus aureus* in pigs in Peru. PLoS ONE. 2011;6:e28529 . 10.1371/journal.pone.0028529 22174831 PMC3234269

[8] Li G, Wu S, Luo W, Su Y, Luan Y, Wang X. *Staphylococcus aureus* ST6-t701 isolates from food-poisoning outbreaks (2006–2013) in Xi’an, China. Foodborne Pathog Dis. 2015;12:203–6 . 10.1089/fpd.2014.1850 25621506

[9] Boost M, Ho J, Guardabassi L, O’Donoghue M. Colonization of butchers with livestock-associated methicillin-resistant *Staphylococcus aureus.* Zoonoses Public Health. 2013;60:572–6. 10.1111/zph.12034 23279691

[10] Bartoloni A, Riccobono E, Magnelli D, Villagran AL, Di Maggio T, Mantella A, Methicillin-resistant *Staphylococcus aureus* in hospitalized patients from the Bolivian Chaco. Int J Infect Dis. 2015;30:156–60. 10.1016/j.ijid.2014.12.006 25486009

