# Supplementary material for: Community-Associated MRSA Infection in Remote Amazon Basin Area, Peru
Source: Emerg Infect Dis. 2016 May;22(5):921–2. doi: 10.3201/eid2205.151881 (PMC4861527; doi:10.3201/eid2205.151881)
Supplement: Supplementary file 1 — Technical Appendix. Pulsed-field gel electrophoresis of known hospital-associated and community-associated methicillin-resistant Staphylococcus aureus isolates from Latin America. [file 15-1881-Techapp-s1.pdf]

Article DOI: http://dx.doi.org/10.3201/eid2205.151881

Community-Associated MRSA Infection in Remote Amazon Basin Area, Peru

Technical Appendix

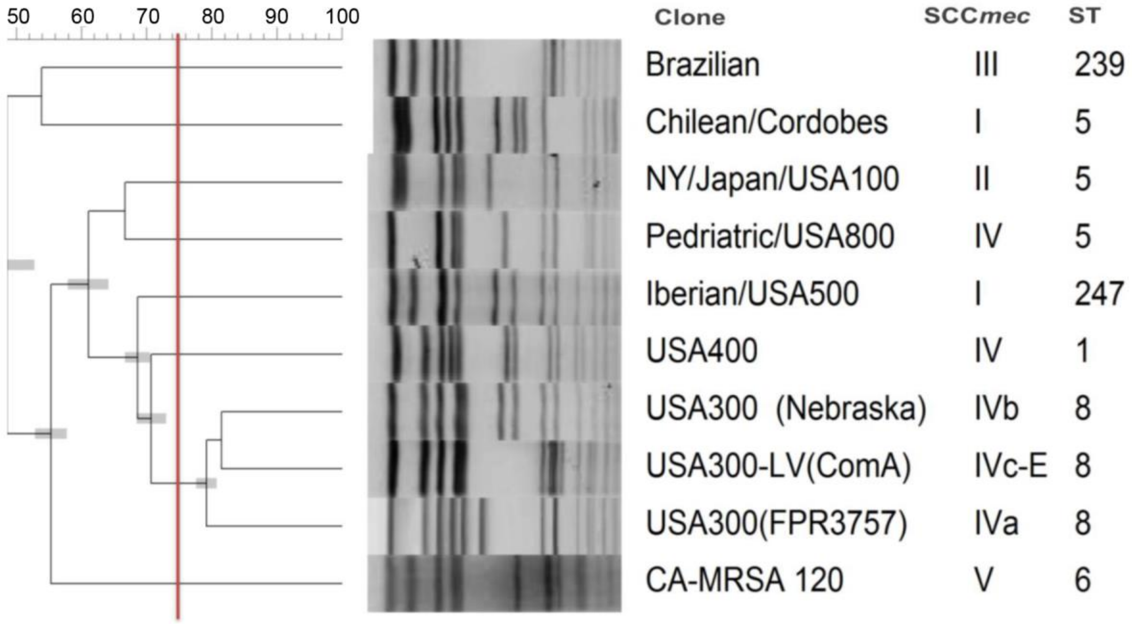

Technical Appendix Figure. Pulsed-field gel electrophoresis of known hospital-associated and community-associated methicillin-resistant *Staphylococcus aureus* (CA-MRSA) isolates from Latin America. Optimization 0.5, tolerance 1, cutoff 75%. SCC, staphylococcal cassette chromosome; ST, sequence type.
